# Unveiling the potential impact of vitamin C in postoperative spinal pain

**DOI:** 10.1186/s41016-024-00368-1

**Published:** 2024-06-01

**Authors:** Fatemeh Ranjbari, Ehsan Alimohammadi

**Affiliations:** 1 Clinical Research Development Center Taleghani and Imam Ali Hospital, Kermanshah University of Medical Sciences, Kermanshah, Iran; 2 Department of Neurosurgery, Kermanshah University of Medical Sciences, Kermanshah, Iran

**Keywords:** Vitamin C, Ascorbic acid, Spinal surgery, Postoperative pain, Review article, Background

## Abstract

**Background:**

Spinal surgery is a common procedure associated with significant postoperative pain, and identifying effective interventions to manage this pain is crucial for optimizing patient outcomes. This review assesses the existing literature to determine the overall impact of vitamin C supplementation on spinal postoperative pain.

Vitamin C, also known as ascorbic acid, is an essential nutrient that plays a vital role in numerous physiological processes. It functions as a potent antioxidant, neutralizing free radicals and reducing oxidative stress within the body. Furthermore, vitamin C is a cofactor in collagen synthesis, a crucial component of connective tissues, including those found in the spinal structures. Given its antioxidant and collagen-promoting properties, vitamin C has piqued interest as a potential therapeutic option for postoperative spinal pain.

Based on the available evidence, vitamin C may have a beneficial effect on postoperative spinal pain, including reducing pain scores, analgesic consumption, and the incidence of complications such as complex regional pain syndrome. However, more research is needed to fully understand the optimal dosage and duration of vitamin C supplementation for postoperative pain management.

**Conclusion:**

Vitamin C could be considered a potentially beneficial adjunctive therapy for managing spinal postoperative pain, but its routine use requires further investigation.

## Background

Spinal surgery is a significant medical intervention often employed to address a wide range of conditions, from herniated discs to spinal stenosis and fractures [1, 2]. While these procedures can greatly improve patients’ quality of life, postoperative pain management remains a crucial aspect of the recovery process [3–5]. In recent years, researchers and healthcare professionals have explored various adjunctive therapies to enhance pain relief and promote faster healing [6–8]. One such intervention that has garnered attention is the administration of vitamin C, renowned for its antioxidant properties and essential role in collagen synthesis. This article delves into the potential impact of vitamin C in postoperative spinal pain, examining the scientific evidence and shedding light on its proposed mechanisms of action [9, 10].

Postoperative spinal pain can manifest as acute discomfort at the surgical site, nerve-related pain, or chronic pain that persists beyond the expected healing period. Traditional approaches to pain management often involve the use of opioids and non-steroidal anti-inflammatory drugs (NSAIDs). However, these medications are associated with various side effects, including addiction potential, gastrointestinal complications, and impaired wound healing. As a result, researchers have sought alternative strategies to minimize patients’ reliance on these pharmaceuticals and their associated risks [11–13].

Vitamin C, also known as ascorbic acid, is an essential nutrient that plays a vital role in numerous physiological processes. It functions as a potent antioxidant, neutralizing free radicals and reducing oxidative stress within the body. Furthermore, vitamin C is a cofactor in collagen synthesis, a crucial component of connective tissues, including those found in the spinal structures. Given its antioxidant and collagen-promoting properties, vitamin C has piqued interest as a potential therapeutic option for postoperative spinal pain [14–17].

Several studies have explored the effects of vitamin C supplementation in surgical patients, focusing on its potential analgesic properties and ability to facilitate wound healing [18–20].

In a randomized, placebo-controlled trial, Lee et al. investigated the efficacy of vitamin C on postoperative outcomes after posterior lumbar interbody fusion [11]. The results of the study revealed several intriguing findings. The authors reported significant improvements in postoperative outcomes, particularly in pain intensity, among the group receiving vitamin C supplementation compared to the placebo group. This finding aligns with previous research suggesting the potential analgesic properties of vitamin C. The authors also reported a reduction in analgesic consumption in the vitamin C group, indicating its potential role in reducing the need for pain medication postoperatively. However, the authors provided limited information regarding the vitamin C dosage and administration route utilized in the study.

In a systematic review and meta-analysis, Sutur et al. evaluated the effects of vitamin C on perioperative outcomes in adults undergoing noncardiac surgery [19]. The authors conducted a comprehensive analysis of 37 randomized controlled trials (RCTs) involving 2747 patients. The primary outcome measured was hospital length of stay (LOS). The results showed no significant difference in LOS between the vitamin C group and the placebo or no treatment group. Similarly, there was no difference in mortality rates between the two groups. However, it is important to note that the evidence supporting these conclusions was of low to moderate quality.

While the study did not find a significant impact on LOS or mortality, several secondary outcomes were explored. Vitamin C administration was associated with a reduction in postoperative pain score and cumulative morphine consumption up to 48 h after surgery. Additionally, orthopedic patients receiving vitamin C had a lower incidence of complex regional pain syndrome. Adverse events were rare but not systematically assessed across the trials, and one trial was stopped early due to safety concerns regarding vitamin C in kidney transplantation surgery.

Mahajan et al. in a prospective study presented compelling evidence regarding the efficacy of vitamin C as an adjuvant in postoperative pain management for patients undergoing surgical decompression in cases of prolapsed intervertebral disc [13].

The prospective study design, which included 50 patients aged 30–60 years, rigorously evaluated the efficacy of vitamin C supplementation as an adjuvant. The randomization of patients into two groups, with one group receiving vitamin C supplementation (group A) and the other group not receiving it (group B), allowed for a comparative analysis of pain outcomes and analgesic requirements.

The results of the study revealed statistically significant differences between the two groups. At the 2nd and 4th-week follow-ups, the mean Numeric Rating Scale (NRS) scores were lower in group A, indicating reduced pain levels compared to group B. Furthermore, the consumption of analgesic medication at the 6-week mark was significantly lower in group A, suggesting a dose-sparing effect of vitamin C supplementation.

These findings highlight the potential of vitamin C as an effective adjuvant in postoperative pain management for patients undergoing spinal decompression surgery. By reducing pain levels and decreasing analgesic requirements, vitamin C supplementation offers a promising avenue to enhance patient comfort and optimize recovery.

The study’s clinical outcome-based approach, coupled with the statistical analysis, strengthens the credibility of the findings. The authors’ conclusion that vitamin C acts as an efficacious adjuvant in postoperative pain management aligns with the observed data and contributes to the existing body of knowledge in this field.

These studies and resources collectively suggest that vitamin C supplementation may have beneficial effects on postoperative pain management, wound healing, and overall surgical outcomes in spine surgeries. However, it is important to consider the limitations of individual studies, such as sample size, study design, and variations in dosing regimens. Further research is needed to establish standardized protocols and guidelines for the use of vitamin C in spine surgeries.

### Mechanisms of action

The proposed mechanisms by which vitamin C may alleviate postoperative spinal pain are multifaceted and involve its antioxidant properties and role in collagen synthesis [6, 9, 13, 17]. Here are some additional details on these mechanisms:

1. Antioxidant effects: vitamin C is a potent antioxidant that helps neutralize free radicals, which are highly reactive molecules that can cause oxidative stress and tissue damage. Postoperative spinal pain is often associated with inflammation and oxidative stress at the surgical site. By reducing oxidative stress, vitamin C may help alleviate pain by minimizing tissue damage and inflammation.
2. Anti-inflammatory effects: in addition to its antioxidant properties, vitamin C has been shown to have anti-inflammatory effects. Inflammation plays a significant role in the development and persistence of postoperative pain. By modulating the inflammatory response, vitamin C may help reduce pain levels and promote healing.
3. Collagen synthesis and tissue repair: vitamin C is an essential cofactor in collagen synthesis, a process crucial for the formation and maintenance of connective tissues, including those in the spinal structures. By promoting collagen synthesis, vitamin C may enhance the structural integrity of spinal tissues, potentially reducing strain on surrounding nerves and alleviating pain. Improved tissue repair and regeneration may also contribute to overall pain reduction and faster recovery.
4. Neuroprotective effects: vitamin C has been implicated in neuroprotective mechanisms. Postoperative spinal pain can involve nerve-related pain, such as radicular pain resulting from nerve compression or irritation. Vitamin C’s antioxidant and anti-inflammatory properties may help protect nerves from damage and reduce pain signals transmitted to the brain.

It is important to note that while these mechanisms are proposed based on the known effects of vitamin C, the specific mechanisms by which vitamin C alleviates postoperative spinal pain are still not fully elucidated. Further research is needed to provide a comprehensive understanding of these mechanisms and their contributions to pain relief.

Moreover, it is worth mentioning that the effectiveness of vitamin C in alleviating postoperative spinal pain may vary among individuals, and optimal dosages and administration methods are still being investigated.

### Specific dosages of vitamin C

The recommended dosage of vitamin C for postoperative pain can vary depending on various factors, including the individual’s overall health, the extent of the surgical procedure, and their specific nutritional needs. It is important to consult with a healthcare professional for personalized advice and to determine the most appropriate dosage for your specific situation [18–21]. However, here are some general guidelines and reference points:

1. Dietary reference intake (DRI): the dietary reference intake for vitamin C varies depending on age, sex, and life stage. For adults, the recommended daily allowance (RDA) is typically around 75–90 mg per day. This amount is intended to meet the basic nutritional needs of the general population and may not specifically target postoperative pain management.
2. Supplementation for wound healing: some studies investigating the effects of vitamin C on postoperative pain and wound healing have utilized higher doses. These doses can range from 500 mg to 2 g per day, often administered intravenously in the immediate postoperative period. However, it is important to note that these studies are specific to certain surgical procedures and patient populations, and the optimal dosage for postoperative spinal pain is not yet well-established.
3. Individualized approach: determining the appropriate dosage of vitamin C for postoperative pain should be done on an individual basis. Healthcare professionals take into account factors such as the patient's overall health, pre-existing conditions, and specific surgical procedures. They can provide personalized recommendations tailored to your needs.

It is important to note that excessive intake of vitamin C beyond the recommended dosages may not necessarily provide additional benefits and could potentially lead to adverse effects, as discussed earlier. Always follow the advice and guidance of your healthcare professional when considering vitamin C supplementation for postoperative pain.

Additionally, it is important to maintain a balanced and nutritious diet that includes a variety of fruits and vegetables, as they are natural sources of vitamin C and other essential nutrients. A well-rounded diet can contribute to overall healing and recovery after spinal surgery.

### Potential side effects

While vitamin C is generally considered safe when taken within recommended dosages, there are potential risks associated with higher-dose vitamin C supplementation in surgical patients. It is important to be aware of these risks and discuss them with your healthcare professional before considering higher-dose vitamin C supplementation [5, 9, 22]. Here are some potential risks:

1. Gastrointestinal upset: higher doses of vitamin C can cause gastrointestinal symptoms such as diarrhea, nausea, and abdominal cramps. These symptoms are generally temporary and resolve when the dosage is reduced. However, they can be problematic for surgical patients who are already experiencing postoperative discomfort and may exacerbate gastrointestinal issues.
2. Kidney stones: there is a theoretical concern that high-dose vitamin C supplementation may increase the risk of kidney stone formation in susceptible individuals. This risk is more relevant to individuals with a history of kidney stones or those with certain medical conditions affecting kidney function. If you have a history of kidney stones or kidney disease, it is important to consult with a healthcare professional before considering higher-dose vitamin C supplementation.
3. Interactions with medications: vitamin C can interact with certain medications. For example, high doses of vitamin C may interfere with the effectiveness of certain chemotherapy drugs and anticoagulant medications, such as warfarin. Additionally, vitamin C may enhance the absorption of iron, which could be problematic for individuals with iron overload disorders. It is crucial to inform your healthcare provider about all the medications you are taking to identify and manage any potential interactions.
4. Allergic reactions: while rare, allergic reactions to vitamin C supplements can occur. Symptoms may include hives, itching, swelling, or difficulty breathing. If you experience any signs of an allergic reaction, discontinue use and seek medical attention.
5. Overall safety profile: while vitamin C is generally well-tolerated, the safety and tolerability of higher-dose vitamin C supplementation in surgical patients are still being investigated. Clinical trials and studies have provided some evidence of potential benefits, but further research is needed to fully understand the risks and benefits in this context.
6. Oxalate accumulation: vitamin C is metabolized in the body to oxalate, a substance that can contribute to the formation of kidney stones. Higher doses of vitamin C can lead to increased oxalate production and potentially raise the risk of oxalate-related kidney stone formation, particularly in individuals with a predisposition to kidney stones. If you have a history of kidney stones or are at risk for developing them, it is important to discuss the potential risks with your healthcare professional.
7. Pro-oxidant effects: while vitamin C is predominantly known for its antioxidant properties, at higher doses it can exhibit pro-oxidant effects. This means that in certain situations, high levels of vitamin C may generate reactive oxygen species (ROS) instead of neutralizing them. This pro-oxidant activity could potentially contribute to oxidative stress and tissue damage, particularly in individuals with certain medical conditions or a delicate balance of antioxidants in their bodies. The clinical significance of these pro-oxidant effects and their impact on surgical patients are still not well understood.
8. Blood clotting: vitamin C can enhance the absorption of iron, which may have implications for individuals at risk of excessive iron accumulation or those with certain blood disorders. In some cases, high-dose vitamin C supplementation has been associated with an increased risk of blood clotting events. If you have a history of blood clotting disorders or are taking medications that affect blood clotting, it is essential to discuss the potential risks with your healthcare professional.
9. Interference with diagnostic tests: high-dose vitamin C supplementation can interfere with certain diagnostic tests, such as glucose or uric acid measurements. This interference can lead to inaccurate results and potentially affect medical decisions. If you are undergoing any diagnostic tests, inform your healthcare provider about your vitamin C supplementation to ensure an accurate interpretation of the results.

It is important to note that the risks associated with higher-dose vitamin C supplementation in surgical patients are still being investigated, and the available evidence is not conclusive. The potential risks may vary depending on individual factors, overall health, and specific surgical procedures. Therefore, it is crucial to consult with a healthcare professional who can assess your individual case and provide personalized advice based on your specific needs and circumstances.

### The role of vitamin C in wound healing and tissue repair following spinal surgery

Vitamin C plays a crucial role in wound healing and tissue repair following spinal surgery. This essential nutrient is required for the synthesis of collagen, a protein that is essential for the formation of connective tissue, skin, tendons, and ligaments. Collagen provides structural support to the body and is necessary for the healing of wounds and the repair of damaged tissues [23, 24].

During spinal surgery, tissues are cut, manipulated, and sutured back together, leading to inflammation and tissue damage. Adequate levels of vitamin C are necessary to support the production of collagen and promote the healing of these tissues. Vitamin C also acts as an antioxidant, protecting cells from oxidative stress and promoting the formation of new blood vessels, which are essential for delivering oxygen and nutrients to the healing tissues [24, 25].

Deficiency in vitamin C can impair wound healing and tissue repair, leading to delayed recovery, increased risk of infection, and poor surgical outcomes. Patients undergoing spinal surgery are at a higher risk of vitamin C deficiency due to the stress of the surgical procedure and the increased metabolic demands of healing.

To support wound healing and tissue repair following spinal surgery, it is important for patients to consume an adequate amount of vitamin C through their diet or supplements. Healthcare providers may recommend vitamin C supplementation to ensure optimal levels of this essential nutrient during the recovery period. Additionally, a balanced diet rich in fruits and vegetables can help provide the necessary nutrients for healing and support overall recovery after spinal surgery [24, 25].

### The role of vitamin C in postoperative pain management and its potential to reduce the need for medication

Vitamin C, also known as ascorbic acid, has been shown to play a role in postoperative pain management and may have the potential to reduce the need for medication following surgery. Pain is a common and expected outcome after surgery, as tissues are damaged and inflammation occurs. Adequate levels of vitamin C are essential for the body’s natural healing processes and can help alleviate pain and discomfort [24, 26].

Vitamin C has antioxidant properties that can help reduce inflammation and oxidative stress, which are key factors in the development of postoperative pain. By neutralizing free radicals and reducing inflammation, vitamin C can help alleviate pain and promote faster healing of tissues. Additionally, vitamin C is involved in the production of neurotransmitters such as serotonin and dopamine, which are important for regulating pain perception and mood [26, 27].

Several studies have shown that vitamin C supplementation can help reduce postoperative pain intensity and the need for pain medication. By supporting the body’s natural healing processes and reducing inflammation, vitamin C can help manage pain more effectively and improve overall recovery outcomes. In some cases, patients who receive adequate levels of vitamin C may require lower doses of pain medication or experience less severe pain following surgery.

It is important for patients undergoing surgery to maintain optimal levels of vitamin C before and after the procedure to support the body’s healing processes and manage postoperative pain. Healthcare providers may recommend vitamin C supplementation as part of a comprehensive pain management plan to help reduce the need for medication and improve the overall recovery experience for patients. Additionally, a balanced diet rich in vitamin C-containing foods such as citrus fruits, berries, and leafy greens can help support pain management and promote faster healing after surgery [6, 27].

### Limitations

Limitations of the current review include the heterogeneity of study designs and methodologies across the included studies. The variability in the dosage, timing, and duration of vitamin C supplementation makes it challenging to draw definitive conclusions about its efficacy in managing postoperative spinal pain. Additionally, the small sample sizes and lack of standardized outcome measures in some studies may introduce bias and limit the generalizability of the findings.

Furthermore, the majority of the studies included in this review focused on short-term outcomes, and there is a lack of long-term follow-up data on the effects of vitamin C supplementation on postoperative pain and recovery. Longitudinal studies with extended follow-up periods are needed to assess the sustained benefits of vitamin C in managing spinal postoperative pain and its impact on long-term outcomes such as functional recovery and quality of life.

Overall, while the existing evidence suggests a potential benefit of vitamin C supplementation in postoperative spinal pain management, the limitations of the current literature highlight the need for well-designed, prospective studies with larger sample sizes, standardized protocols, and longer follow-up periods to establish the efficacy and optimal use of vitamin C as an adjunctive therapy for postoperative pain.

## Conclusions

Vitamin C supplementation has emerged as a potential adjunctive therapy for postoperative spinal pain. Its antioxidant properties and role in collagen synthesis suggest the potential for pain relief and improved healing outcomes. While promising, it is important to note that the existing body of research on the impact of vitamin C in postoperative spinal pain is still relatively limited. Further well-designed clinical trials are necessary to establish optimal dosages, identify potential contraindications or adverse effects, and elucidate the precise mechanisms by which vitamin C exerts its pain-modulating effects.

## Data Availability

All data generated or analyzed during this study are included in this published article.

## References

[1] Chaitanya NC, Muthukrishnan A, Krishnaprasad C, Sanjuprasanna G, Pillay P, Mounika B (2018). An insight and update on the analgesic properties of vitamin C. J Pharm Bioallied Sci.

[2] Connolly D, Lauzon C, Agnew J, Dunn M, Reed B (2006). The effects of vitamin C supplementation on symptoms of delayed onset muscle soreness. J Sports Med Phys Fitness.

[3] Daoust R, Paquet J, Chauny J-M, Williamson D, Huard V, Arbour C (2023). Impact of vitamin C on the reduction of opioid consumption after an emergency department visit for acute musculoskeletal pain: a double-blind randomised control trial protocol. BMJ Open.

[4] Fukushima R, Yamazaki E (2010). Vitamin C requirement in surgical patients. Curr Opin Clin Nutr Metab Care.

[5] Hung K-C, Lin Y-T, Chen K-H, Wang L-K, Chen J-Y, Chang Y-J (2020). The effect of perioperative vitamin C on postoperative analgesic consumption: a meta-analysis of randomized controlled trials. Nutrients.

[6] Jain SK, Dar MY, Kumar S, Yadav A, Kearns SR (2019). Role of anti-oxidant (vitamin-C) in post-operative pain relief in foot and ankle trauma surgery: a prospective randomized trial. Foot Ankle Surg.

[7] Jensen NH (2003). Reduced pain from osteoarthritis in hip joint or knee joint during treatment with calcium ascorbate. A randomized, placebo-controlled cross-over trial in general practice. Ugeskrift for Laeger.

[8] Kanazi GE, Yazbeck-Karam VG, Hanna JE, Masri B, Aouad MT (2012). Effect of vitamin C on morphine use after laparoscopic cholecystectomy: a randomized controlled trial. Can J Anesth.

[9] Kaźmierczak-Barańska J, Boguszewska K, Adamus-Grabicka A, Karwowski BT (2020). Two faces of vitamin C—antioxidative and pro-oxidative agent. Nutrients.

[10] Kiabi FH, Soleimani A, Habibi MR, Zeydi AE (2013). Letters to Editor: can vitamin C be used as an adjuvant for managing postoperative pain? A short literature review. The Korean J Pain.

[11] Lee GW, Yang HS, Yeom JS, Ahn MW (2017). The efficacy of vitamin C on postoperative outcomes after posterior lumbar interbody fusion: a randomized, placebo-controlled trial. Clin Orthop Surg.

[12] Lu R, Kallenborn-Gerhardt W, Geisslinger G, Schmidtko A (2011). Additive antinociceptive effects of a combination of vitamin C and vitamin E after peripheral nerve injury. PLoS ONE.

[13] Mahajan NP, Narvekar MA, Chandanwale AS, Gadod LL, Kumar GP (2021). Vitamin C supplementation as adjuvant analgesic therapy in post-operative pain management in patients undergoing surgical decompression in a case of prolapsed intervertebral disc. International Journal of Research in Medical Sciences.

[14] Rice ME (2000). Ascorbate regulation and its neuroprotective role in the brain. Trends Neurosci.

[15] Rosa KA, Gadotti VM, Rosa AO, Rodrigues ALS, Calixto JB, Santos AR (2005). Evidence for the involvement of glutamatergic system in the antinociceptive effect of ascorbic acid. Neurosci Lett.

[16] Shah AS, Verma MK, Jebson PJ (2009). Use of oral vitamin C after fractures of the distal radius. Journal of Hand Surgery.

[17] Shibuya N, Humphers JM, Agarwal MR, Jupiter DC (2013). Efficacy and safety of high-dose vitamin C on complex regional pain syndrome in extremity trauma and surgery—systematic review and meta-analysis. J Foot Ankle Surg.

[18] Smith VH (2010). Vitamin C deficiency is an under-diagnosed contributor to degenerative disc disease in the elderly. Med Hypotheses.

[19] Suter M, Pinto BB, Belletti A, Putzu A (2022). Efficacy and safety of perioperative vitamin C in patients undergoing noncardiac surgery: a systematic review and meta-analysis of randomised trials. Br J Anaesth.

[20] Zollinger P, Tuinebreijer W, Breederveld R, Kreis R (2007). Can vitamin C prevent complex regional pain syndrome in patients with wrist fractures?: A randomized, controlled, multicenter dose-response study. JBJS.

[21] Pacier C, Martirosyan DM (2015). Vitamin C: optimal dosages, supplementation and use in disease prevention. Functional Foods in Health and Disease.

[22] Vojdani A, Bazargan M, Vojdani E, Wright J (2000). New evidence for antioxidant properties of vitamin C. Cancer Detect Prev.

[23] Uehara H, Itoigawa Y, Morikawa D, Koga A, Tsurukami H, Maruyama Y (2023). The effect of vitamin C and N-acetylcysteine on tendon-to-bone healing in a rodent model of rotator cuff repair. Am J Sports Med.

[24] Moores J (2013). Vitamin C: a wound healing perspective. Br J Community Nurs.

[25] Bikker A, Wielders J, Van Loo R, Loubert M (2016). Ascorbic acid deficiency impairs wound healing in surgical patients: Four case reports. Int J Surg Open.

[26] Carr AC, McCall C (2017). The role of vitamin C in the treatment of pain: new insights. J Transl Med.

[27] Chen S, Roffey DM, Dion C-A, Arab A, Wai EK (2016). Effect of perioperative vitamin C supplementation on postoperative pain and the incidence of chronic regional pain syndrome: a systematic review and meta-analysis. Clin J Pain.

